# The outcome of rituximab in treating steroid dependent nephrotic syndrome. Histopathology and immunosuppressive drugs as predicting factors

**DOI:** 10.15537/smj.2022.43.7.20210727

**Published:** 2022-07

**Authors:** Abdullah A. Al Salloum, Adi J. Al Herbish, Mohammed A. Al Hissi, Mohammed S. Abdalla, Suha B. Salim, Afrah H. Farhat, Reem A. Shagal, Abduldafaee Othman, Abdulelah Alshaiban, Mohamad-Hani A. Temsah, Ayman A. Al-Eyadhy, Khalid A. Alhasan

**Affiliations:** *From the Department of Pediatrics (Al Salloum, Al Herbish, Al Hissi, Abdallah, Salim, Farhat, Shagal, Othman, Alshiban, Temsah, Al-Eyadhy, Alhasan), College of Medicine, King Saud University, King Saud University Medical City, and from the Department of Pediatric Kidney Transplant (Alhasan), Organ Transplant Center of Excellence, King Faisal Specialist Hospital and Research Center, Riyadh, Kingdom of Saudi Arabia.*

**Keywords:** rituximab, nephrotic syndrome, children, steroid-dependent

## Abstract

**Objectives:**

To present our experience of treating steroid-dependent nephrotic syndrome (SDNS) in children with repeated doses of rituximab (RTX) with a relatively long follow-up, and to discuss the role of the histopathology type and previous immune-suppressor (IS) drugs on the outcome of these patients.

**Methods:**

The patients included in this prospective study were children with SDNS who were in remission on a high-dose steroid or with additional IS drugs. All patients underwent renal biopsy before RTX treatment. Intravenous RTX was administered monthly at 375 mg/m^2^ for 4 doses. Response to treatment was defined as maintaining remission with no steroid-sparing agents or prednisone for one year.

**Results:**

Seventeen (14 males) patients were enrolled. Approximately 76% had minimal change disease (MCD) and 3 (18%) patients had immunoglobulin M (IgM) nephropathy. Approximately 85% of MCD and 33% of IgM nephropathy showed complete response to RTX.

**Conclusion:**

Compared to other IS used to treat SDNS, RTX showed a significant decrease in relapse rate with fewer side effects. The dose and interval should be modified according to the patient’s characteristics, such as medical history, pathology type, and previous IS agents.

**K**idney Disease Improving Global Outcome 2012 suggested a low-dose alternative day steroid as the first-line treatment for the management of steroid-dependent nephrotic syndrome (SDNS).^1^ Prolonged use of steroids in growing children has many adverse effects, including short stature, diabetes mellitus, cataracts, susceptibility to infections, and others.

Additional immunosuppressive agents are often required to minimize the adverse effects of steroids. Alkylating agents (such as cyclophosphamide, calcineurin inhibitors [CNIs]) and antiproliferative agents have been used.^2^ None of these alternatives have many adverse effects.^3^ These drugs need regular monitoring, and there is a great burden on patients and their families as these drugs need to be administered regularly on a daily basis for months or years and are not always effective. Other side effects should always be considered, such as the risk of infections, nephrotoxicity, hypertension, cosmetic, gonadal toxicity, and risk of malignancy.^3^

There is a real need for a relatively safe drug that does not require frequent administration. In recent years, rituximab (RTX) has been shown to be an efficient treatment for idiopathic nephrotic syndrome (INS) and more precisely for SDNS, especially in patients who relapse despite maintenance IS with CNI or mycophenolate mofetil (MMF).^4,5^ Kidney Disease Improving Global Outcome 2012 suggested that RTX can be considered in children with SDNS who continue to relapse despite the use of steroids and steroid-sparing agents.^6^ Also, there are few studies that showed its efficacy and safety.^7,8^

Rituximab has become popular for the treatment of INSs, and some issues should be considered. First, some patients may relapse while on CD20-B-cell depletion, or even undetectable. There is also a need to use another parameter, such as renal histology, to determine which patient may benefit from single or frequent doses of RTX. Renal histology as a predictor of the efficacy of RTX therapy has been infrequently addressed in the literature. Second, the safety of long-term B-cell suppression caused by repeated administration of RTX in children, whose immune systems are still developing, remains unknown.

The role of previous IS drugs on the response to RTX needs to be assessed. Herein, we present our experience of treating SDNS in children with repeated doses of RTX with a relatively long follow-up, and we discussed the role of the histopathology type and previous IS drugs on the outcome of these patients.

## Methods

This prospective study was carried out between 2009-2019 at King Saud University Medical City, Riyadh, Saudi Arabia, to demonstrate the efficacy of RTX in children with SDNS and to examine the impact of histopathology and previous IS agents on long-term response.

The study was approved by the Research and Ethical Committee of the College of Medicine, King Saud University (project no. E-09-034). Informed consent forms were signed by the patients’ parents after face-to-face interviews with the treating physician.

Patients included in this study were children (age: 4-13 years) with nephrotic syndrome that were dependent on steroids for more than one year with manifestation of drug toxicity or use of steroids and other IS drugs to maintain remission. Patients who did not follow up regularly or lost follow up and those who got secondary steroid resistance were excluded.

Additionally, renal biopsy was carried out to determine the histopathological type and to rule out CNI evidence of nephrotoxicity. Steroid-dependent nephrotic syndrome was defined as a 2 consecutive relapses during weaning or within 2 weeks of steroid withdrawal. High steroid dependency was defined as the requirement of prednisolone of >0.5 mg/kg on alternate days to maintain remission. Relapse was defined as morning proteinuria of >2+ for 3 consecutive days.

### Treatment protocol of RTX

Rituximab was administered during remission of nephrotic syndrome at a monthly intravenous dose of 375 mg/m^2^ for 4 doses. Our initial protocol was to administer 4 doses; however, in 2017, we encountered serious side effects in patients with sustained hypogammaglobulinemia that required the administration of monthly intravenous immunoglobulins. The protocol modification after that date was left for the treating physician to give either a single dose and observe or to give the initial course of 4 doses. In the long-term follow-up, we found that most patients required more than 4 doses. Two or more additional doses were administered after relapse.

To reduce the risk of infusion reaction, the patients received 1 mg/kg intravenous methylprednisolone one hour before RTX infusion.

For patients who were on CNI or MMF, slow withdrawal over 4 months was recommended. In patients who relapsed during the 4 months period of RTX, intravenous methylprednisolone (10 mg/kg/day for 3 days) was administered to achieve urinary remission before administering the next dose of RTX. Prior to each dose of RTX, clinical and laboratory parameters, such as renal function, serum albumin, urine albumin to creatinine ratios, serum immunoglobulin level, and CD19 + B-cell count were carried out. B-cell depletion was defined as a CD19 count of < 1% of the total lymphocytes, and B-cell recovery was defined as a >2%. Response to treatment was defined as patient is off medications for minimum of one year. Partial response was defined as a decrease in the number or dose of the previous drugs by 50% to maintain remission. Result data were collected and analyzed by simple counting average and percentage.

### Statistical analysis

All statistical analyses were carried out using the Statistical Package for the Social Science, version 25.0 (IBM Corp., Armonk, NY, USA). The frequencies and percentages were used to represent all categorical variables.

## Results

A total of 17 patients with SDNS, comprising 14 (82%) male, with mean age of 8 years (4-13 years), were enrolled. All patients underwent renal biopsy prior to RTX, and no patients had features suggestive of nephrotoxic drug effects on kidney pathology. The results of the renal biopsy revealed minimal change disease (MCD) in 13 (76%) of the patients, one (6%) with focal segmental glomerulosclerosis (FSGS), and 3 (18%) with mesangial proliferation with immunoglobulin M (IgM) deposition. The mean duration of the disease prior to commencement of RTX therapy was 4.6 years (range: 2-9 years). The follow-up period from the last dose of RTX ranged between 2-10 years (average 3.9 years). The number of doses administered to each patient ranged from 2-12 doses (average 4.9 doses). The detailed clinical characteristics of the patients are presented in Table 1.

**Table 1:** - Patients’ clinical characteristics.

No.	Gender	Onset age (y)	Previous IS	Kidney biopsy	Age at RTX (y)	No. of RTX infusion	Adverse effect	Time since last infusion (y)	Last visit urine dipstick	Outcome and comments
1	F	4	Pred, tac	MCD	9	6	No	2	- ve	Remission, off medication
2	M	3	Pred, MMF	MCD	7	8	No	3	trace	Remission, off medication
3	F	4	Pred, CyA	MCD	11	6	Bronchiectasis Hypogamma-globulinemia	3	- ve	Remission, on monthly IV IgG replacemen
4	F	5	Pred	MCD	12	4	No	10	- ve	Remission, off medication for 10 years
5	M	6	Pred, CyA, MMF	MCD	10	12	Anaphylactoid reaction to last dose	2	4+	Pred, MMF, Tac
6	M	4	Pred, MMF	MCD	9	4	No	5	- ve	Remission, off medication for 5 years
7	M	2	Pred, CyA	MCD	4	4	No	3	- ve	Remission, on low-dose pred
8	M	3	Pred, MMF, CyA	IgM Nephropathy	6	2	No	2	- ve	Remission, on MMF
9	M	2	Pred, MMF	IgM Nephropathy	4	4	No	3	- ve	Remission, off medication
10	M	5	Pred, MMF	MCD	10	3	No	9	- ve	Remission, off medication for 9 years
11	M	4	Pred, MMF	MCD	8	3	No	8	- ve	Remission, off medication for 8 years
12	M	6	Pred, CyA	MCD	12	7	No	3	- ve	Remission, off medication
13	M	4	Pred, CyA	MCD	6	2	No	2	Trace	Remission, off medication
14	M	3	Pred, MMF, Tac	IgM Nephropathy	5	3	Allergy, itching	2	- ve	Remission on MMF
15	M	2	Pred, CyA	FSGS	4	2	No	2	- ve	Remission on CyA
16	M	2	Pred	MCD	10	4	No	2	- ve	Remission, off medication
17	M	4	Pred, CyA	MCD	13	4	No	5	- ve	Remission, off medication for 5 years

No.: number, y: years, IS: immunosuppressive medication, RTX: rituximab, F: female, M: male, Pred: prednisolone, tac: tacrolimus, MMF: mycophenolate mofetil, CyA: cyclosporine A, MCD: minimal change disease, IgM: immunoglobulin M, FSGS: focal segmental glomerulosclerosis, IgG: immunoglobulin G, IV: intravenous

All patients who were on steroids (2 patients) or steroid plus MMF (5 patients) responded to RTX; none of these patients were on any medications during 2-10 years of follow-up. A total of 7 patients received steroids and CNI prior to RTX, and 5 were off medication after 2-5 years of follow-up. Two of the 3 patients who had 3 IS medications showed a partial response, while one still needed 3 drugs to maintain remission (Table 2).

**Table 2:** - Outcomes according to pre-rituximab medication.

Pre-RTX drug	No. of patients	Response	Partial response	No response
Steroid alone	2	2	0	0
Steroid + MMF	5	5	0	0
Steroid + CNI	7	5	2	0
Steroid + MMF +CNI	3	0	2	1

RTX: rituximab, No.: number, MMF: mycophenolate mofetil, CNI: calcineurin inhibitors

Of the 13 patients who had MCD, 11 (85%) had complete response and were off medication for 2-10 years; 3 of these patients were off medications for 8, 9, and 10 years. One patient showed a partial response initially but became unresponsive after 4 years.

Three (18%) patients had IgM nephropathy; one responded well and remained off therapy for 3 years after the 4^th^ dose, while one patient who was on triple IS medications was maintained on MMF and carried out well. We could not administer the 4^th^ dose because of severe itching that he developed after the third dose of RTX. The third patient also received a combined therapy and received 2 RTX doses only and was maintained on single IS drugs. One (6%) patient with FSGS received 2 doses of RTX; he was weaned from steroids and has been maintained on CNI for the last 2 years (Table 3).

**Table 3:** - Outcomes according to histopathology.

Pathology	No. of patients	Response	Partially response	No response
MCD	13 (76.0)	11/13	1/3	1/13
IgM nephropathy	3 (18.0)	1/3	2/3	0
FSGS	1 (6.0)	No	1/1	0
Total	17 (100)	12/17	4/17	1/17

Values are presented as number and precentage (%). No.: number, MCD: minimal change disease, IgM: immunoglobulin M, FSGS: focal segmental glomerulosclerosis

A total of 14 (82%) patients tolerated RTX very well, with no immediate or late adverse reactions. A 12-year-old female patient developed recurrent chest infections and bronchiectasis with persistent hypogammaglobulinemia after the 6^th^ dose of RTX. She was in complete remission for over 3 years, but unfortunately, she required regular intravenous immunoglobulin infusion on a monthly basis. Another patient experienced anaphylactic reactions 15 minutes after RTX infusion. The third patient with IgM nephropathy experienced itching after the 3^rd^ dose, which prevented the continuation of RTX treatment.

## Discussion

The standard treatment for SDNS in children is IS medication, including cyclophosphamide, levamisole, MMF, or CNI, in addition to the lowest possible dose of steroids.^9^ These medications are not always effective because of the risk of side effects, particularly nephrotoxicity. The need to give these medications on a regular basis daily for months or even years puts a heavy burden on patients and their families.

In 2004, Benz et al^10^ reported the case of a patient with complicated nephrotic syndrome that was resistant to treatment, who developed refractory idiopathic thrombocytopenic purpura (ITP); this patient was treated with RTX (375 mg/m^2^) once weekly for 4 consecutive weeks without any adverse events, and both ITP and nephrotic syndrome resolved. Many reports and studies have suggested that RTX is a promising drug for the treatment of difficult nephrotic syndrome. However, the number of doses, dose interval, strength of the dose, and use of adjunct IS therapy remain controversial.^2,9,11^

In our cohort we tested the pathological type of nephrotic syndrome to determine which patients may benefit more from RTX therapy; our results are encouraging and agree with the findings of most published studies. In addition, we assessed the effect of the number of IS before RTX therapy on the patients’ response to RTX.

We observed that the patients who were on steroids alone or steroids and MMF had the best outcome with long-term remission after RTX. Patients on steroid and CNI showed less favorable outcomes, while none of the patients on a combination of steroids, MMF, and CNI had full response, and one had no response and continued receiving combined IS medication despite a mild histopathology MCD and B-cell depletion. Ravani et al^12^ showed that patients receiving low doses of steroids alone responded very well to RTX.

In our study, we observed that patients receiving multiple IS drugs showed less response to RTX. The influence of histological subtype on the response of patients to RTX is unclear. The effect of RTX on MCD compared to FSGS in previous studies showed that there was a higher probability of non-response in patients with FSGS.^13^

Immunoglobulin M nephropathy is a relatively less-recognized clinico-immunopathological entity that is characterized by diffuse mesangial deposition of IgM, with approximately one-third of cases being steroid dependents, with the remaining two-thirds being steroid resistant or high-dose dependent.^14^ We could not find a study that discussed the effect of RTX on IgM nephropathy. In our study, we had 3 patients with IgM nephropathy, one of whom had complete remission, and the other 2 had partial response to RTX.

The optimal dose of RTX has not yet been established. The trial using 100 mg/m^2^ versus 375 mg/m^2^ versus 750 mg/m^2^ has been discussed in the literature; however, most of the studies used a dose of 375 mg/m^2^.

Maxted et al^2^ found no significant difference in B-cell recovery and duration of remission between a dose of 750 mg/m^2^ and that of 375 mg/m^2^. Hogan et al^15^ found that a dose of 375 mg/m^2^ was better than a dose of 100 mg/m^2^ as it had fewer cases of relapses and longer B-cell depletion. We used a dose of 375 mg/m^2^ as it was almost universally agreed upon.

The frequency of RTX administration varies among studies. Hogan et al^15^ found that 2 doses were better than a single dose in ensuring better B-cell depletions. Takahashi et al^16^ concluded that administering RTX 4 times may be excessive in patients who had no relapses within one year after the initial RTX administration. Our initial protocol until 2017, was to administer 4 doses; however, we faced serious side effects in patients with sustained hypogammaglobulinemia that required the administration of monthly intravenous immunoglobulins. After this encounter, the modification of the protocol (whether to give a single dose and observe or to give the initial course of 4 doses) was left to the discretion of the treating physician. In the long-term follow-up, we found that most patients required more than 4 doses.

The duration between doses was almost unified in previous studies on weekly intervals for the first doses, which was based on the oncology experience; this suggests that MCD is a benign disease compared to non-Hodgkin’s lymphoma, and the risk of progression to stage 5 chronic kidney disease is rare. We used monthly instead of weekly doses of RTX to reduce side effects. We found that the efficacy of our monthly doses was comparable to that of weekly doses.

### Study limitations

One was generated from a single center, in addition a small sample size. A further prospective multicenter study with large sample size is recommended.

In conclusion, compared to other IS agents used to treat SDNS, RTX has a significantly lower relapse rate with fewer side effects. Rituximab reduces the burden on patients and their families. The dose strength and interval should be modified according to the patients’ characteristics, such as medical history, pathological type, and use of previous IS agents.
